# Editorial: Methods in breast cancer

**DOI:** 10.3389/fonc.2023.1304941

**Published:** 2023-11-10

**Authors:** Gianluca Franceschini

**Affiliations:** Fondazione Policlinico Universitario Agostino Gemelli IRCCS, Breast Unit, Department of Woman and Child Health and Public Health, Università Cattolica del Sacro Cuore, Rome, Italy

**Keywords:** breast cancer, new methods & methodologies, surgery, oncoplastic breast conserving surgery, mastectomy, breast reconstruction, breast treatment, radiotherapy

Breast cancer remains one of the most prevalent challenges in oncology and is recognized as an international priority in healthcare; it is currently the most common tumor in women worldwide with demographic trends indicating a continued increase in incidence (1). While clinicians and researchers work to find an optimal strategy in treatment of breast cancer, the introduction of innovative methods is also mandatory.

Nowadays, therapeutic strategies against breast cancer are increasingly personalized for each patient. The appropriate treatment is modulated on clinical characteristics, staging, biological factors such as the status of hormone receptors, Ki67, HER2 overexpression. In particular, endocrine therapy plays a vital role in the management of estrogen receptor-positive breast cancer. Chen et al. examine how clinicopathological factors influence decisions regarding extended endocrine therapy. They report that that age, lymph nodal status and receipt of chemotherapy are independent predictors for the recommendation of extended endocrine therapy.

However, a multidisciplinary management is essential to improve oncological and aesthetic results while increasing patient’s quality of life. An accurate discussion should always be carried out with each patient on the benefits and problems related to the chosen treatment.

Accurate coding is crucial for an appropriate disease classification and research; Tu et al. review discrepancies in ICD-9/ICD-10-based codes for breast cancer and other common diseases, highlighting the need for precision in health data. They indicate that researchers should use standardized, validated coding algorithms to reduce risk of misclassification which can significantly alter the findings of a study.

Thanks to the wider diffusion of screening programs, breast cancer is more often detected at an early stage. An innovative ultrasound-based radiomics model can distinguish between sclerosing adenosis and invasive ductal carcinoma while potentially aiding in early diagnosis (Huang et al.). This innovative model can contribute to an effective diagnosis by avoiding misdiagnosis and unnecessary biopsies.

Early-stage breast cancer is usually treated with primary surgery. Breast-conserving treatment or mastectomy are the surgical options. However local control of disease and the patient’s aesthetic satisfaction should always be guaranteed in both cases (1). An adequate evaluation of the disease by clinical and radiological assessment is mandatory to choose the best local treatment (1). The selection of optimal surgical treatment should be based on breast volume, cancer size, multicentricity, ability to obtain clear surgical margins and the patient’s wishes.

Breast-conserving surgery (BCS) with adjuvant radiotherapy is considered the gold standard approach for early-stage breast cancer. Some prospective randomized studies have not shown significant differences in disease-free and overall survival rates comparing conservative treatment with mastectomy. However, BCS must always guarantee complete surgical removal of the tumor with negative surgical margins and optimal aesthetic results also using some biomaterials as filler (2, 3). Nowadays, the main method used to evaluate margins in BCS is a pathological evaluation. The designation of surgical margins is controversial and metabolomics may represent a new approach to assess surgical margins. Based on metabolomic analysis, Wang et al. show the negative margin of 1 mm is sufficient for surgery. The six metabolites identified as abnormal (pyruvic acid, N-acetyl-L-aspartic acid, glutamic acid, gamma-aminobutyric acid, fumaric acid and citric acid) may serve as biomarkers to select an appropriate surgical margin.

Adjuvant radiotherapy is a cornerstone after BCS. However, subsequent cardiac toxicity is deemed to be dose-dependent for left breast cancer irradiation. Chen et al. investigate the impact of respiratory capacity on dose sparing during left-sided breast cancer irradiation, highlighting a novel technique for optimizing radiotherapy. They demonstrate the effect of respiratory capacity for dose sparing when the deep inspiration breath hold with Active Breathing Coordinator technique (ABC-DIBH) is used in left-sided breast cancer irradiation. Furthermore, Dabbs et al. present the results on clinical utility for the DCISionRT test for the prediction of recurrence risk and radiotherapy benefit in ductal carcinoma *in situ*.

Mastectomy should be performed when conservative treatment is unable to guarantee adequate local control and appropriate aesthetic results. Common indications for mastectomy include: large tumors that cannot be treated by BCS with a satisfactory cosmetic outcome; multicentric disease; persistent positive margins after multiple re-excisions; inability to perform adjuvant radiotherapy; presence of BRCA pathogenic variants; patient preference (1). Breast reconstruction after mastectomy is a critical aspect of a patient’s recovery. Immediate breast reconstruction with autologous tissue or prosthesis should always be performed after mastectomy as it can improve the patient’s quality of life (4). The demand for further aesthetic improvement in breast reconstruction is leading to innovative solutions. Lee et al. delves into the clinical outcomes of robot-assisted DIEP (Deep Inferior Epigastric artery Perforator) flap surgery. They suggest that robotic DIEP flap offers enhanced postoperative recovery with a reduction in postoperative pain and hospital stay.

Regarding axillary surgery, sentinel lymph node biopsy is considered the gold standard in early breast cancer with clinically negative nodes. Axillary dissection is indicated in breast cancers with clinically positive nodes although new therapeutic strategies are emerging (1). Lei et al. report a nomogram for predicting positive non-sentinel lymph nodes (non-SLNs) in positive SLN patients.

Primary chemotherapy is used with increasing frequency in the multidisciplinary treatment of breast cancer patients (5). Various trials have demonstrated that neoadjuvant chemotherapy allows to obtain important advantages such as downstaging of disease favoring surgical de-escalation (6, 7). Wang et al. propose a multimodal approach to predict clinical responses to neoadjuvant therapy in advanced breast cancer, incorporating B-mode ultrasonography, shear wave elastography and pathological information.

The primary aim of management in metastatic breast cancer is to mitigate symptoms, prolong survival and improve quality of life. Patients with metastatic disease can be treated with endocrine therapy, chemotherapy, biologic therapies and immunotherapy (1). HER2-positive patients with brain metastases respond favorably to pyrotinib and trastuzumab-based treatment (Chen et al.). Zhang et al. report the efficacy and safety of apatinib (an oral small-molecule tyrosine kinase inhibitor targeting VEGFR-2) 250 mg combined with chemotherapy in patients with pretreated metastatic breast cancer. Yang et al. explore the clinicopathological characteristics of bone marrow metastases in breast cancer and prognosis using different therapies. Ippolito et al. show the results of the BOMB trial, which explores the use of stereotactic radiotherapy on primary tumor in metastatic patients (20). The study evaluates the maximum tolerated dose of stereotactic body radiotherapy (SABRT) to primary breast cancer in stage IV disease.

Furthermore, some authors take us into personalized medicine by demonstrating how patient-derived explant cultures can recapitulate *in vivo* drug responses, opening the way to innovative treatments (Pettersen et al.). Finally, innovative trials and new technologies are changing breast cancer management; single-arm designs with non-inferiority and superiority analyses are optimal for proof-of-concept and de-escalation studies in oncology (Sampayo-Cordero et al.). The telehealth care is also an adequate approach to reduce the treatment burden and the clinical problems of breast cancer survivors (Ajmera et al.).

In conclusion, this Research Topic presents interesting research in the field of breast cancer treatment. Thanks to results of ongoing studies and collaboration between healthcare professionals and researchers, prognosis and well-being of breast cancer survivors can be constantly improved.

## Author contributions

GF: Conceptualization, Writing – original draft, Writing – review & editing.
